# Tandem RNA and Protein Extraction: A Platform for Maximizing the Use of Limited Ex Vivo Tissue Samples

**DOI:** 10.21769/BioProtoc.5643

**Published:** 2026-03-20

**Authors:** Ciarán Kennedy, Braden Millar, Luke J. Conroy, Mariam Marai, Mary Barry, Martin O’Donohoe, Orina Belton, Eoin Brennan, Catherine Godson, Monica de Gaetano

**Affiliations:** 1 Diabetes Complications Research Centre, Conway Institute and School of Biomolecular and Biomedical Science, University College Dublin, Ireland; 2 Diabetes Complications Research Centre, Conway Institute and School of Medicine, University College Dublin, Ireland; 3 Department of Vascular Surgery, St. Vincent's University Hospital, Dublin, Ireland; 4 Mater Misericordiae University Hospital, Cardiovascular Surgery Department, Dublin, Ireland

**Keywords:** RNA extraction, Protein isolation, Atherosclerosis, Human carotid endarterectomy, RNA*later*, Tandem RNA and protein isolation, Patient samples

## Abstract

Human tissue samples represent the gold standard for obtaining clinically relevant and translatable insight into disease processes that in vitro systems cannot fully reproduce. However, patient-derived samples are often limited in size and availability, limiting the number of downstream assays that can be performed. To maximize the use of invaluable human samples, we present a protocol for the tandem extraction of high-quality RNA and protein from the same tissue section. This method has been optimized for 15–30 mg tissue sections, enabling more experimental conditions and technical replicates, while minimizing intrasample variability associated with heterogeneous tissues. This protocol also avoids potentially hazardous solvents present in phenol-chloroform-based methods such as TRIzol, providing a safer and more accessible workflow without compromising biomolecule integrity. This protocol was developed and validated using atherosclerotic plaque tissue from carotid endarterectomy, a very challenging tissue type to work with due to extensive calcification, necrosis, and limited surgical availability. We have also validated this method using mouse aortic tissue and cultured THP-1 cells, demonstrating its versatility across sample input types. As this protocol relies on standard column-based RNA extraction kits and commonly available reagents for protein precipitation and extraction, this methodology is widely accessible and easy to implement as a standard, streamlined workflow.

Key features

• Advances ex vivo models of human disease (e.g., atherosclerosis) by maximizing the use of patient-derived tissue samples.

• Minimizes patient variability by generating directly comparable RNA and protein readouts.

• Minimizes the number of patient samples required and maximizes the output from each sample.

• Stabilizes RNA integrity from tissue without impacting the quality of proteins derived from the same sample.

## Graphical overview


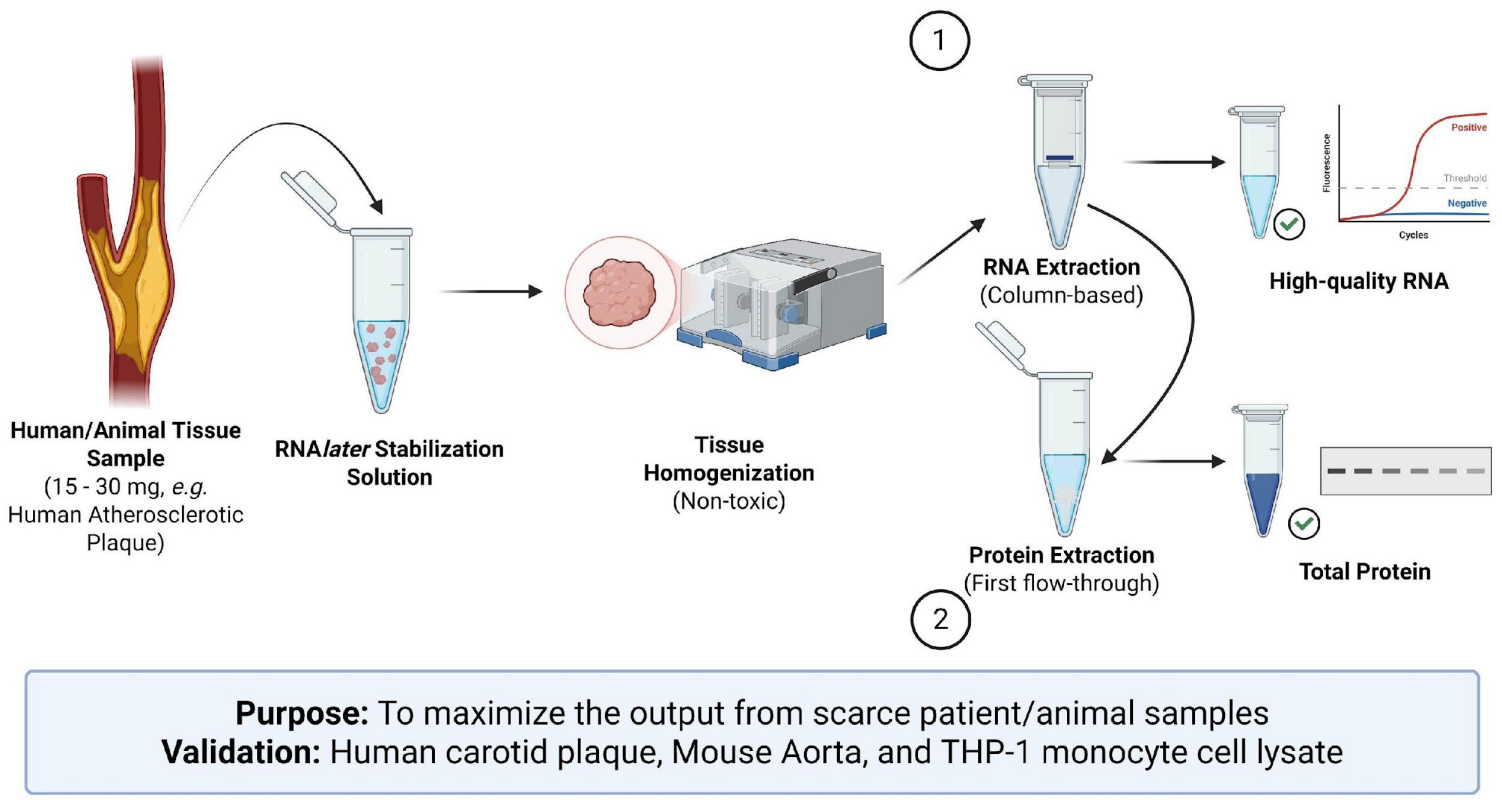


## Background

Human tissue samples provide strong clinically relevant insight into disease processes that in vitro and in vivo systems cannot fully replicate. However, they pose some experimental challenges, such as heterogeneity and limited sample availability. Similarly, in vitro models, including cultured 3D models, possess their own limitations, as they do not recreate the integrated physiological environment of human disease. Animal models remain essential for mechanical and therapeutic studies, but translational issues often arise due to species-specific differences. For example, though human and murine macrophage subtypes share broad functional similarities, they diverge in important clinically relevant pathways, such as species-specific activation patterns of the PPARγ pathway [1]. Therefore, patient-derived tissue remains the most reliable source of directly translatable information.

The limited size and availability of patient samples restrict the number of downstream analyses that can be performed. This scarcity means that researchers often must be strategic about which assays to perform, given the limited number of samples available. To address this challenge, this protocol enables the tandem extraction of high-quality RNA and protein from the same tissue sample. This approach maximizes the usage of valuable human specimens while reducing intrasample variability, which may arise from sampling heterogeneous tissues.

This method is optimized for small tissue sections (15–30 mg), allowing more experimental conditions and technical replicates to be included. The use of RNA*later* stabilizes RNA and protein, preserving integrity for one week at room temperature, one month at 4 °C, or indefinitely at -20 °C or lower [2]. Unlike common commercial methods like TRIzol, this workflow avoids toxic organic solvents like phenol and chloroform.

We developed and validated this protocol primarily with ex vivo–treated atherosclerotic plaque tissue from carotid endarterectomy (CEA). This tissue type provides substantial challenges because carotid plaques are often highly calcified due to long-term inflammation, necrosis, mechanical stress, aging, and osteogenic pathways, which are hallmarks of atherosclerotic disease. CEA tissue is also often limited in size and surgical availability. The use of small 15–30 mg (3 × 3 × 3 mm) sections for both RNA and protein could address some of these limitations. These small pieces of carotid tissue also remain metabolically active when cultured ex vivo for at least 24 h and are reactive to stimuli over this timeframe. This allows for the ex vivo treatment and screening of compounds of interest directly in human atherosclerotic tissue. The possibility of using small pieces of tissue and extracting both protein and RNA from them maximizes experimental outputs.

In addition to human CEA samples, we confirm the suitability of this protocol for use in mouse aortic tissue and THP-1 cells, and we expect this workflow to be adaptable to a wide range of tissue and sample types with minimal optimization (primarily in tissue volume and homogenization), because it relies on standard column-based RNA extraction kits and commonly available laboratory reagents. As a result, the protocol is widely accessible and compatible with most laboratory settings, providing an opportunity to maximize experimental readouts in most settings and a cheaper alternative to commercially available kits such as the Allprep (Qiagen) and Ambion (Thermo Fisher Scientific) kits. It also provides a layer of RNA stabilization, negating the need for liquid nitrogen snap freezing and tissue grinding for RNA extraction from tissue.

While several protocols have been described for precipitating total protein from various sources, including RNA extraction kit flowthrough [3–6], the protocol described here offers several distinct advantages. It avoids the use of hazardous organic compounds such as trichloroacetic acid, acetone, methanol, or chloroform. By avoiding these harsh organic compounds and avoiding washing with 96%–100% ethanol (as described in [6]), this protocol avoids stripping the proteins completely of hydration, facilitating easier downstream solubilization. Furthermore, by using only the first flowthrough of the RNA-extraction procedure for protein precipitation, samples can be processed in smaller volumes and tube sizes, reducing reagent and plasticware consumption.

## Materials and reagents

**Biological materials**

1. Human tissue sample, e.g., carotid endarterectomy (approximately 3 × 3 × 3 mm in size or 15–30 mg in weight)

2. Animal tissue sample, e.g., snap-frozen mouse aorta (tissue samples of 15–30 mg, including small organs, can be processed whole if the tissue thickness does not exceed 5 mm)

3. Cell lysate, e.g., THP-1 monocytes

**Reagents**

1. β-mercaptoethanol, 100 mL (Sigma-Aldrich, catalog number: M3148)

2. Laemmli buffer and DTT, 8 mL (Cell Signaling Technology, catalog number: 7722S)

3. Ethanol 200-proof (Fisher Scientific, catalog number: CCE7023)

4. TRK lysis buffer (EZNA Extraction Kit) (Omega BIO-TEK, catalog number: R6834-02)

5. For tissue samples: Invitrogen RNA*later* stabilization solution, 100 mL (ThermoFisher, catalog number: AM7020) (fresh or ex vivo–treated samples) or Invitrogen RNA*later*-ICE tissue transition solution (ThermoFisher, catalog number: AM7030) (snap-frozen samples)

**Solutions**

1. 70% ethanol solution (see Recipes)

2. TRK lysis buffer with 2% β-mercaptoethanol (see Recipes)

**Recipes**

**1. 70% ethanol solution**

**Table:** 

Reagent	Final concentration	Quantity or volume
Ethanol	70%	70 mL
Ultrapure water	-	30 mL

Store at 4 °C.

**2. TRK lysis buffer with 2% β-mercaptoethanol**

**Table:** 

Reagent	Final Concentration	Quantity or volume
TRK lysis buffer (or equivalent)	-	9.8 mL
β-mercaptoethanol	2%	200 µL

Store at 4 °C. Stable for one month.

**Laboratory supplies**

1. Column-based total RNA extraction kit (e.g., EZNA Total RNA Kit I, Omega BIO-TEK, catalog number: R6834-02)

2. 1.5 mL centrifuge tubes (Greiner, catalog number: 616201CIS)

3. 2 mL homogenizer tubes and caps (Sarstedt, catalog numbers: 72.609 and 65.716.004, respectively)

4. 50 and 15 mL Falcon tubes (Greiner, catalog numbers: 210270CIS and 188261CIS, respectively)

5. Pipette filter tips (P2–P1000)

6. Serological pipette strips (P5–P25)

7. 5 mm stainless steel beads (Qiagen, catalog number: 69989)

## Equipment

1. Refrigerated centrifuge with a relative centrifugal force of at least 14,000× *g*

2. Bead homogenizer (QIAGEN, model: TissueLyser II)

3. Heat-block capable of 100 °C (Eppendorf, model: ThermoMixer_F2.0)

4. Fridge (4 °C)

5. Freezer (-20 °C and -80 °C)

6. Falcon tube and centrifuge tube racks

7. Nanodrop spectrophotometer (DeNovix DS-11 Spectrophotometer)

8. Pipettes (P2–P1000)

9. Stainless-steel tweezers (Merck, catalog number: 22435-U)

10. Scales (capable of measuring with milligram-level accuracy)

## Procedure

The amounts, times, and volumes listed below were optimized for the specific RNA extraction kit, homogenization equipment, and tissue types used in this study. For other sample types, please follow the instructions of the specific RNA extraction kit and homogenizer being used.

**A. Sample processing**

**A1. For ex vivo–treated tissue samples**

1. Place tissue sections into a labeled 1.5 mL centrifuge tube containing 300 μL of pre-chilled RNA*later* and store at 4 °C at least overnight

2. The following day, aspirate off the RNA*later*.

3. Proceed immediately to homogenization of tissue or store tissue for later use at -20 °C or below.

**A2. For previously frozen tissue samples**

1. Place frozen tissue sections into a labeled 1.5 mL centrifuge tube containing 300 μL of pre-chilled RNA*later*-ICE solution and incubate at -20 °C for at least 16 h.

2. For storage, keep the samples in RNA*later*-ICE and store at -20 °C or below. When ready to process, aspirate off the RNA*later*-ICE and proceed immediately to homogenization. Samples are stable in RNA*later*-ICE for up to 30 min at room temperature.

*Notes:*

*1. Duplicate/triplicate tissues can be grouped together and placed in the same solution of RNA*later *without changing the volume of RNA*later *in the tube, as the existing volume is sufficient to cover the tissue.*

*2. RNA in RNA*later*-treated samples is stable at 4 °C for one month and at -20 °C (or lower) indefinitely.*

*3. RNA in samples treated with RNA*later*-ICE is stable for at least 16 h at 4 °C and at -20 °C (or lower) indefinitely (as long as the tissue remains submerged in RNA*later*-ICE).*

*4. The use of RNA*later*-ICE can dry out the snap-frozen tissue, making accurate determination of tissue weight difficult.*

**A3. For cultured THP-1 cells**

1. Collect up to 5 × 10^6^ cells by centrifugation and remove residual medium or PBS by aspiration.

2. Add 350 μL of TRK lysis buffer, supplemented with 2% β-mercaptoethanol, mix thoroughly, and incubate at room temperature for 5 min.

3. Add an equal volume of 70% ethanol and proceed to section C.

**B. Tissue homogenization**

1. Weigh out the tissue in a homogenizer tube (optional) before adding a stainless-steel homogenizer bead and 350 μL of TRK lysis buffer with 2% β-mercaptoethanol.

*Notes:*

*1. Pre-chill TRK lysis buffer with 2% β-mercaptoethanol at 4 °C for at least 30 min before use.*

*2. Add lysis buffer with β-mercaptoethanol under the chemical hood.*

*3. The blocks from the homogenizer can be chilled at -80 °C before use if desired (optional).*

2. Homogenize the tissue in a QIAGEN TissueLyser II for 10 min at 25 oscillations per second.

*Note: Ensure the tissue is fully homogenized before continuing to step B3. If necessary, return to the homogenizer and repeat step B2.*

3. Transfer the homogenized tissue in the lysis buffer into a 1.5 mL centrifuge tube and centrifuge at 12,000 g for 5 min at 4 °C to collect cell debris.

4. Transfer the supernatant into a new, pre-labeled centrifuge tube.

*Note: Some samples may contain more fat and debris than others. A second centrifugation is recommended if you are unable to aspirate off the supernatant without disturbing the pellet, as the debris can clog the RNA extraction column membrane.*

5. Add an equal volume of chilled 70% ethanol to the transferred supernatant and mix thoroughly (final volume of approximately 700 μL).

**C. RNA extraction**

*Note: If using a different column-based RNA extraction kit, follow the manufacturer's instructions for RNA isolation and retain the initial column flowthrough for protein extraction. Some optimization may be required to collect additional wash flowthroughs or adjust the volume of ethanol used for protein precipitation.*

1. Transfer the entire homogeneous solution (approximately 700 μL), including any precipitate that may have formed, into an RNA extraction column placed inside a collection tube and centrifuge at 10,000× *g* for 1 min at 4 °C.

*Note: Retain the initial flowthrough from step C1, as it will be used in section D.*

2. Move the column to a new 2 mL collection tube, add 500 μL of wash buffer I from the EZNA extraction kit, and centrifuge at 10,000× *g* for 30 s at 4 °C.

*Note: Pre-chill the RNA extraction reagents (wash buffer I and II, 70% ethanol, and RNase-free water) at least 30 min before use.*

3. Discard the flowthrough and add 500 μL of wash buffer II before centrifuging at 10,000× *g* for 1 min at 4 °C.

4. Repeat step C3.

5. Centrifuge the spin column at maximum speed (≥14,000× *g*) for 2 min at 4 °C in a clean 2 mL collection tube to dry the spin column membrane.

6. Transfer the spin column to a 1.5 mL centrifuge tube and then add 20–40 μL of RNase-free water to the membrane.

7. Centrifuge at maximum speed for 2 min at 4 °C.

8. (Optional) Collect the eluted RNA and add it back to the membrane again before centrifuging at maximum speed for 2 min at 4 °C again.

*Note: This step can help increase the RNA concentration in the eluted sample and ensure that all RNA is eluted from the column.*

9. Place the centrifuge tube containing the eluted RNA on ice and quantify the RNA concentration using a nanodrop spectrophotometer or store at -80 °C.

**D. Protein extraction**

1. Transfer the flowthrough obtained in step C1 into a pre-labeled 1.5 mL centrifuge tube and add an equal volume of ice-cold 100% ethanol.

2. Invert or vortex the tube until the solution is homogeneous.

3. Store the labeled tube upright at -20 °C for at least 1 h (or preferably overnight) to precipitate the protein.

4. Briefly vortex the sample (2–3 s) to break up any large chunks of precipitate, which may prevent future resuspension.

5. Centrifuge precipitated protein at 2,000× *g* for 3 min at 4 °C.

6. Carefully decant or pipette off the supernatant without disturbing the pellet (a small volume of supernatant can be left behind to avoid pellet disruption).

7. Fully resuspend the pellet in 500 μL of ice-cold 70% ethanol, being careful to include any precipitate that may have settled on the wall of the tube.

*Note: During this step, the pellet must be fully resuspended to ensure that the samples will dissolve later.*

8. Centrifuge resuspended protein at 2,000× *g* for 3 min at 4 °C.

9. Fully resuspend the pellet in 500 μL of ice-cold 70% ethanol again.

10. Centrifuge resuspended protein at 5,000× *g* for 5 min at 4 °C.

11. Remove as much supernatant as possible and allow residual 70% ethanol to evaporate.

*Note: Air dry or use a vacuum aspirator inside the lid of the tube, not touching the sample, to evaporate the ethanol. Take care not to over-dry, as this will prevent proper dissolution of the protein pellet. Do not use a Speed-vac or other vacuum concentrator.*

12. Dissolve the pellet in an appropriate buffer for downstream applications, e.g., for a western blot, 1× Laemmli buffer with DTT. Samples may require heating and/or vortexing to be fully dissolved.

13. Proceed with protein quantification or store for downstream applications (-80 °C).

*Note: Suitable assays for protein quantification will depend on the composition of the final buffer in which the sample is dissolved. For example, the standard Bradford assay is incompatible with detergents (e.g., 1% SDS or Laemmli buffer), while the BCA assay is incompatible with reducing agents (e.g., DTT or 2-mercaptoethanol). The Bio-Rad’s detergent-compatible Bradford assay is compatible with moderate concentrations of detergent and reducing agent.*

## Data analysis

RT-PCR data were assessed by using the Design and Analysis software (version 2.6.0) by Applied Biosystems, and Ct values were converted to a quantitative value relative to the vehicle control samples. Data were normalized to GAPDH expression.

Statistical comparisons were performed by running either a 2-way ANOVA with Sidak’s multiple comparisons or an unpaired T-test on sample sets with a minimum of 4 biological replicates using GraphPad Prism software (version 10.4.2). Statistical significance was set at p < 0.05. Data were expressed as mean relative quantity ± SEM.

Protein concentration was determined using the detergent-compatible Bradford reagent assay. Relative expression of individual targets was quantified by normalizing expression to total protein stain, using the LICOR Image Studio 6 software.

## Validation of protocol

**Figure 1.**
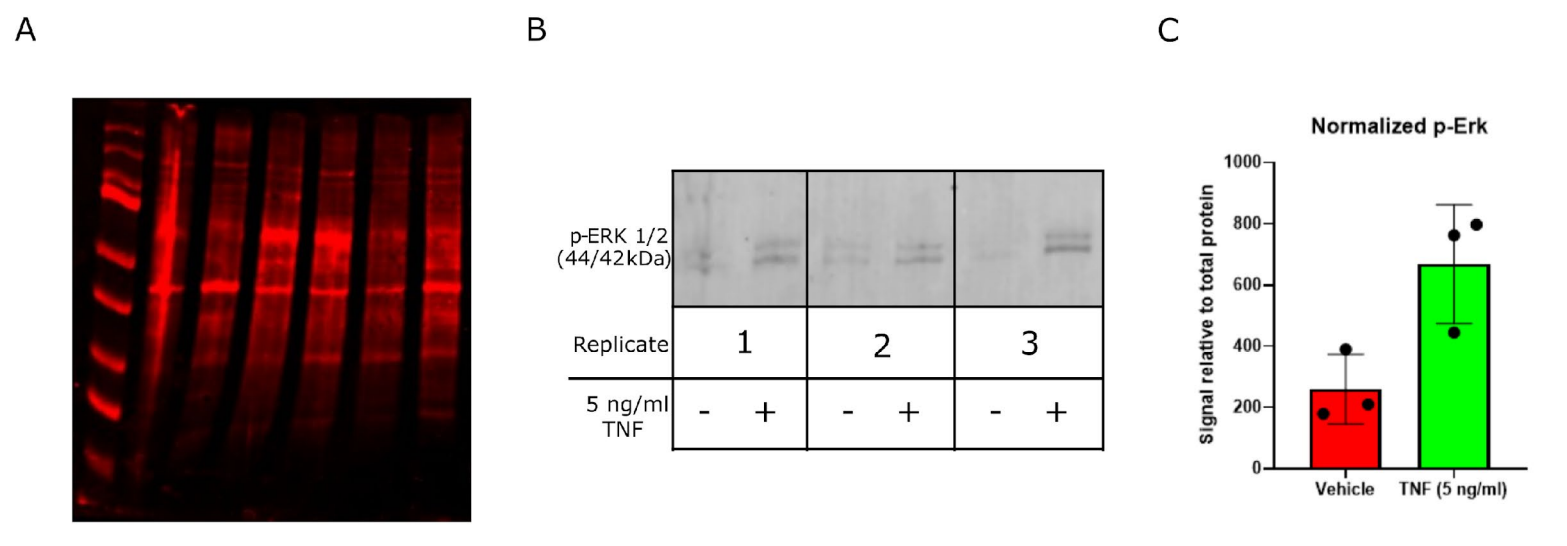
Assessment of protein integrity and responsiveness of ex vivo–treated human carotid plaque tissue. Plaques were stimulated with 5 ng/mL TNF for 24 h, after which both protein and RNA were extracted and analyzed. (A) Fluorescent total protein staining, showing suitable protein distribution and integrity. Proteins were resolved on a 4%–12% SDS-PAGE gel and transferred onto a PVDF membrane using an Invitrogen PowerBlotter system. Following transfer, total protein was stained using the reversible total protein stain Revert 700. Representative imaging was acquired using the LICOR Odyssey CLX imaging system. (B) Membranes were incubated with a primary antibody against phosphorylated p44/p42 MAPK (Erk1/2) (Cell Signaling Technology, #9101) and developed using the LICOR Odyssey CLX imaging system following incubation with an appropriate fluorescently tagged secondary antibody (DyLight 680 conjugated anti-rabbit, Invitrogen, #35569). TNF stimulation increased phosphorylation of ERK1/2 by western blot in plaque tissue after 24 h, indicating tissue viability, responsiveness to external stimuli, and suitability for downstream molecular analyses. Each replicate represents tissue from an individual patient's coronary endarterectomy. (C) Quantification of (B), performed using LICOR Image Studio 6 and normalizing the signal intensity of the protein of interest to the total protein signal from the corresponding lane on the same blot. Presented as mean relative signal ± SEM, n = 3.

**Figure 2.**
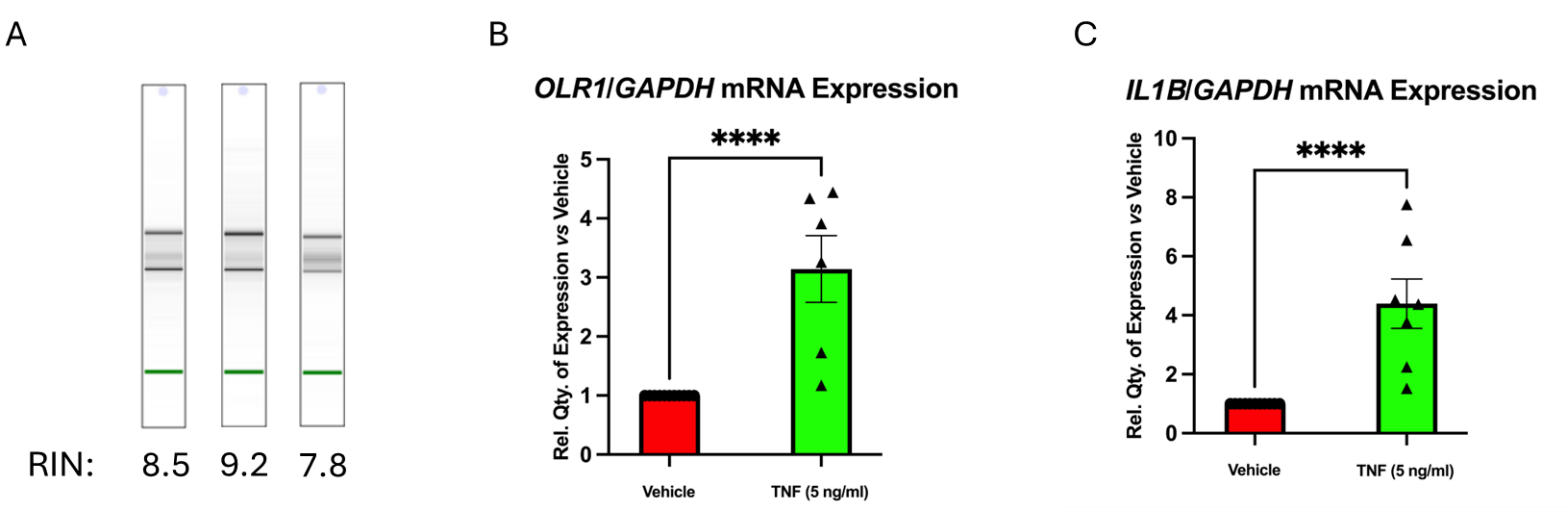
Assessment of RNA integrity and gene expression responsiveness of ex vivo–treated human carotid plaque tissue. Plaques were stimulated with 5 ng/mL TNF for 24 h, after which both protein and RNA were extracted and analyzed. (A) Representative “gel-like” images from Agilent Bioanalyzer analysis showing distinct bands for 18S and 28S rRNA, and RIN (RNA integrity number) values, confirming RNA integrity. (B, C) Expression level of *OLR1* (B) and *IL1B* (C) by qRT-PCR, showing the responsiveness of the tissue to TNF stimulation and suitability for downstream molecular analyses. Statistical analyses were performed using an unpaired two-tailed t-test with data normalized to GAPDH and represented as mean relative quantity ± SEM (n ≥ 6). P ≤ 0.05 was considered statistically significant.

**Figure 3.**
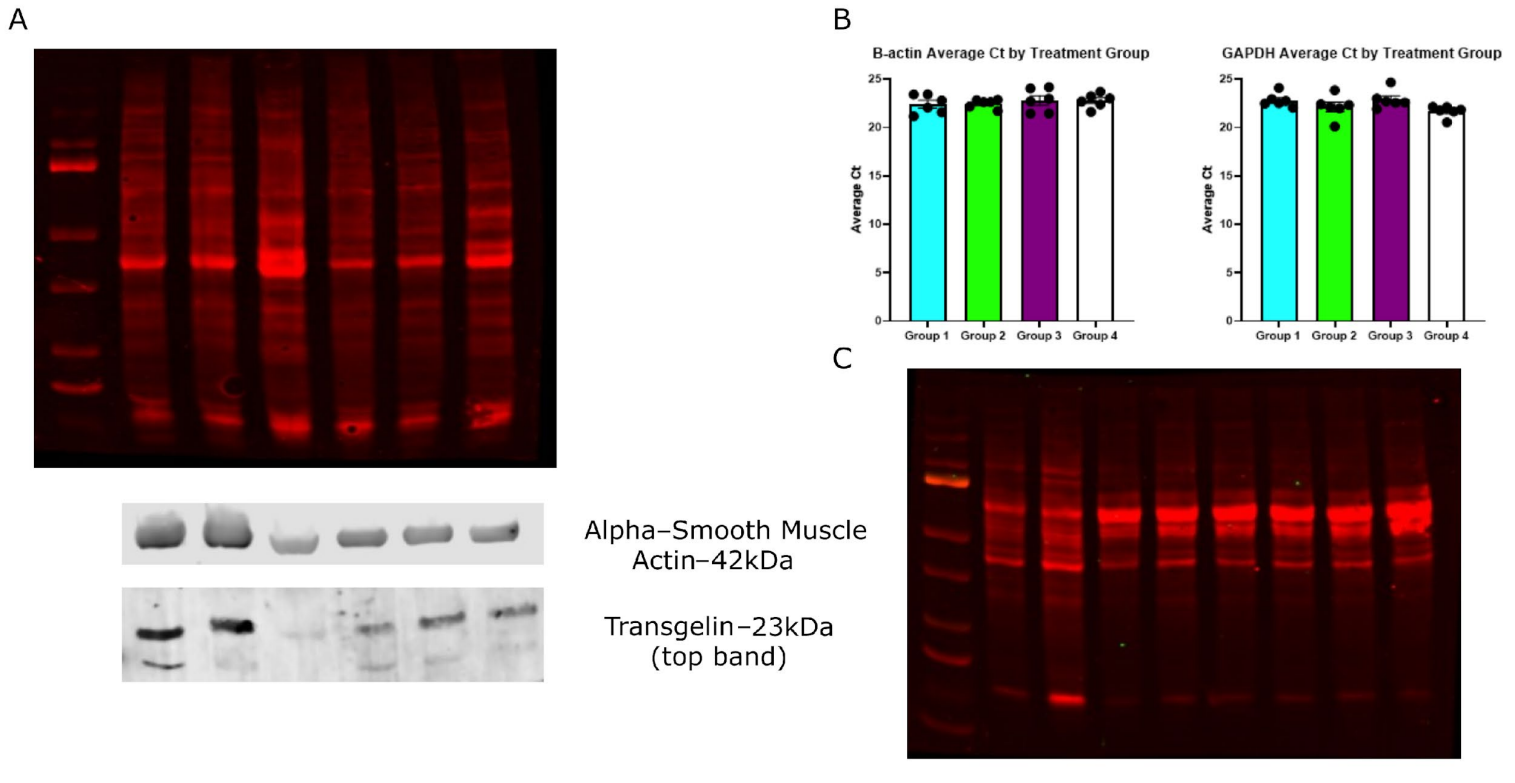
Assessment of RNA and protein integrity from other sources. RNA and proteins were extracted from dissected ApoE-/- mouse aortas (A, B), as well as from THP-1 monocytes and macrophages (C). (A) Fluorescent total protein staining showing suitable protein distribution and integrity using protein extracted from mouse aortas, with representative western blots for alpha-smooth muscle actin (ACTA2) and transgelin (TAGLN). Proteins were resolved on a 4%–12% SDS-PAGE gel and transferred onto a PVDF membrane using an Invitrogen PowerBlotter system. Following transfer, total protein was stained by using the reversible total protein stain Revert 700 and imaged using a LICOR Odyssey CLX imaging system. Membranes were incubated with a primary antibody against alpha-smooth muscle actin (Cell Signaling Technologies, #12945) or Transgelin (Cell Signaling Technology, #40471) and developed using the LICOR Odyssey CLX imaging system following incubation with an appropriate fluorescently tagged secondary antibody (DyLight 680 conjugated anti-rabbit, Invitrogen, #35569). (B) Representative qRT-PCR data for GAPDH and beta-actin show comparable Ct values across samples. Statistical analyses were performed using a one-way ANOVA with Tukey’s multiple comparisons test. Data expressed as the average Ct value of duplicate samples, and represented as mean relative quantity ± SEM (n = 6). P ≤ 0.05 was considered statistically significant. (C) Fluorescent total protein staining shows suitable protein distribution and integrity using protein extracted from cultured THP-1 cells.

To validate the robustness of this protocol, we performed tandem RNA and protein extraction from human CEA tissue (n ≥ 12) (Figures 1–2), ApoE-/- mouse aorta tissue (n ≥ 12) (Figure 3A), and THP-1 monocytes and macrophages (n ≥ 12) (Figure 3B, C). Each replicate of CEA tissue, mouse aorta, and cell lysates was analyzed using an RNA bioanalyser to assess RNA quality. The precipitated proteins were subsequently analyzed by western blotting; total protein staining was performed to evaluate sample quality and usability. Representative examples are displayed here.

## General notes and troubleshooting

**Troubleshooting**

**Problem 1:** Incomplete homogenization of the tissue sample.

Possible causes: Extensive calcification of the tissue.

Solution: Repeat the homogenization step for another cycle of 10 min at 25 oscillations per second. The RNA*later* solution stabilizes the RNA to avoid sample degradation at room temperature. If the tissue cannot be fully homogenized due to extensive calcification of the tissue, continue with any non-calcified tissue portions that have been successfully homogenized (proceed to step B3).

**Problem 2:** Incomplete dissolution of precipitated proteins.

Possible cause (i): Large aggregates have formed during initial precipitation.

Solution: Vortex the initial precipitate thoroughly to break up any large crystals/aggregates that may have formed.

Possible cause (ii): Complete pellet resuspension not achieved after each centrifugation step.

Solution: Ensure that the pellet is fully resuspended before initial centrifugation and after each centrifugation step.

Possible cause (iii): Pellet has been over-dried prior to dissolution in the final buffer.

Solution: Air-dry pellets until the edges just begin to become opaque; do not allow them to fully dry. Do not use a SpeedVac or other vacuum concentrator to remove residual 70% ethanol.

Possible cause (iv): Unsuitable buffer or insufficient volume.

Solutions:

a. Choose a suitable buffer for protein dissolution compatible with your downstream application. Usually, buffers containing a chaotropic agent (e.g., urea or guanidine hydrochloride) or a detergent (e.g., SDS, Triton X-100, or CHAPS) are needed to fully dissolve the pellet.

b. If compatible with the buffer used and downstream application, samples may be heated to ensure dissolution.

*Note: Avoid heating proteins in urea-containing buffers as this accelerates urea decomposition into cyanate, causing irreversible carbamylation of the proteins.*

**Problem 3:** Low quality/amount of extracted RNA.

Possible causes: (i) Overly calcified or necrotic starting tissue; (ii) incorrect handling or storage of starting tissue.

Solutions:

a. Process artery tissue samples as soon as possible after surgical removal to ensure good viability. Samples can be temporarily stored in saline for transport, but should be processed promptly for treatment, storage (ideally in a stabilizing solution, e.g., RNA*later*), or RNA and protein extraction.

b. Avoid overly calcified or necrotic areas of tissue when dissecting samples.

c. Treat multiple small pieces of tissue per condition to increase the likelihood of extracting RNA of suitable quality and quantity for downstream applications.

RNA yield and quality depend on the degree of calcification or necrosis in the starting tissue and on how samples are handled and stored before processing. Storage in RNA*later* helps limit degradation. Where yield or quality is low, using additional technical replicates can help obtain usable material.
